# Influenza A viral loads in respiratory samples collected from patients infected with pandemic H1N1, seasonal H1N1 and H3N2 viruses

**DOI:** 10.1186/1743-422X-7-75

**Published:** 2010-04-20

**Authors:** Nathamon Ngaosuwankul, Pirom Noisumdaeng, Pisut Komolsiri, Phisanu Pooruk, Kulkanya Chokephaibulkit, Tawee Chotpitayasunondh, Chariya Sangsajja, Charoen Chuchottaworn, Jeremy Farrar, Pilaipan Puthavathana

**Affiliations:** 1 Department of Microbiology, Faculty of Medicine Siriraj Hospital, Mahidol University, Bangkok 10700, Thailand; 2 Pediatrics, Faculty of Medicine Siriraj Hospital, Mahidol University, Bangkok 10700, Thailand; 3 Queen Sirikit National Institute of Child Health, Bangkok 10400, Thailand; 4 Bamrasnaradura Infectious Disease Institute, Nonthaburi 11000, Thailand; 5 Chest Disease Institute, Nonthaburi 11000, Thailand; 6 Hospital for Tropical Diseases, Wellcome Trust Major Overseas Programme, Oxford University Clinical Research Unit, Ho Chi Minh City, Vietnam

## Abstract

**Background:**

Nasopharyngeal aspirate (NPA), nasal swab (NS), and throat swab (TS) are common specimens used for diagnosis of respiratory virus infections based on the detection of viral genomes, viral antigens and viral isolation. However, there is no documented data regarding the type of specimen that yields the best result of viral detection. In this study, quantitative real time RT-PCR specific for *M *gene was used to determine influenza A viral loads present in NS, NPA and TS samples collected from patients infected with the 2009 pandemic H1N1, seasonal H1N1 and H3N2 viruses. Various copy numbers of RNA transcripts derived from recombinant plasmids containing complete *M *gene insert of each virus strain were assayed by RT-PCR. A standard curve for viral RNA quantification was constructed by plotting each Ct value against the log quantity of each standard RNA copy number.

**Results:**

Copy numbers of *M *gene were obtained through the extrapolation of Ct values of the test samples against the corresponding standard curve. Among a total of 29 patients with severe influenza enrolled in this study (12 cases of the 2009 pandemic influenza, 5 cases of seasonal H1N1 and 12 cases of seasonal H3N2 virus), NPA was found to contain significantly highest amount of viral loads and followed in order by NS and TS specimen. Viral loads among patients infected with those viruses were comparable regarding type of specimen analyzed.

**Conclusion:**

Based on *M *gene copy numbers, we conclude that NPA is the best specimen for detection of influenza A viruses, and followed in order by NS and TS.

## Background

Influenza A viruses are classified into 16 hemagglutinin (H) and 9 neuraminidase (N) subtypes [1]. Since the emergence of Russian influenza A (H1N1) in 1977 [2] to the emergence of pandemic influenza A (H1N1) in April 2009, only A/H1N1, A/H3N2 and influenza B viruses have been recognized as human or seasonal influenza. Influenza virus spreads via respiratory secretion. After an incubation period of about 1-3 days, the viruses are shed from various kinds of respiratory samples. Upper respiratory tract specimens, such as nasopharyngeal wash (NPW) or nasopharyngeal aspirate (NPA), nasal swab (NS), throat swab (TS), endothracheal swab, bronchoalveolar lavage and tissues, are recommended for virus detection in patients with respiratory tract infection. These specimens could be used for viral antigen detection, virus isolation and molecular methods for genome detection. Nevertheless, there is no documented data which addresses the type of specimen that gives the best yield for the disease diagnosis [3].

Genomes of influenza A and B viruses are composed of 8 negative sense, single-stranded RNA segments encoded for 10-11 proteins essential for infection and replication [1]. The genomic RNA has been used as targets for amplification by conventional and real time reverse transcription-polymerase chain reaction (RT-PCR). The highly conserved *M *gene-derived primers are usually utilized for diagnosis of all influenza A subtypes, whereas specific subtype identification targets *H *or *H *and *N *genes. In this study, the protocol established by the U.S., Center for Disease Control (CDC) for detection of *M *gene [4] in adjunct with the standard curves of known copies of *M *RNA transcripts derived either from H1N1, H3N2 or the 2009 pandemic A (H1N1) viruses was used to quantify the viral loads in specimens collected from patients with severe influenza prior to receiving anti-viral drug. Our study provided the information on the clinical specimens that yielded the best diagnostic result; and the viral loads in patients infected with different influenza subtypes and strains were also compared.

## Methods

### Subjects and Specimen Collection

This study was approved by the Institutional Review Boards of the Committee on Ethics, Faculty of Medicine Siriraj Hospital, Mahidol University and the Ministry of Public Health, Thailand. NPA, NS and TS samples were collected in viral transport medium (MicroTest™ Multi-Microbe Media; Remel, Lenexa, KS) from patients with severe influenza. The collection of NPA was performed by flushing through a nasopharyngeal tube with 2 ml of sterile normal saline using a sterile NG-tube or sterile butterfly needle tube, inserted through the floor of nose. The NPA yield at approximately 0.5 ml volume was then added with VTM and the 3.5 ml final volume was obtained. The nose and throat swabbing were performed right after the NPA collection from nostrils and throat, respectively, using MicroTest™ kit with 3 ml of VTM.

### Quantitative Real time Reverse Transcription-Polymerase Chain Reaction

Real time RT-PCR protocols established by CDC as well as viral antigen detection by QuickVue (Quidel Corporation, San Diego, CA), virus isolation in MDCK cell culture and serodiagnosis, were used to diagnose influenza virus infection in these patients. Positive results from at least two diagnostic tests were obtained for each case. A total of 29 patients enrolled in this study comprised 12 cases of pandemic influenza A/2009 (H1N1), 5 cases of A/Brisbane/59/2007(H1N1) like- and 12 cases of A/Brisbane/10/2007 (H3N2) like-virus infection. All respiratory specimens were kept at -70°C until tested.

In the preparation of standard *M*-RNA, viral RNA extracted from A/Nonthaburi/102/2009 (H1N1), A/Brisbane/59/2007-like (H1N1) and A/Brisbane/10/2007-like (H3N2) viruses were reverse transcribed into complementary DNA (cDNA) in a 20 μl reaction comprised 8 μl of viral RNA, 1× RT buffer, 5 mM MgCl_2_, 10 mM DTT, 50 ng of random hexamers, 0.5 mM dNTPs, 40 units of RNaseOUT^TM^ (Invitrogen Corporation, Carlsbad, CA) and 200 units of SuperScript^TM^ III reverse transcriptase (Invitrogen) following the manufacturer's instruction. Thereafter, cDNA was subjected to PCR amplification in a 50 μl reaction mixture containing 5 μl of cDNA target, 5 μl of 10× High Fidelity PCR buffer, 1 mM dNTP mixture, 2 mM MgSO_4_, 0.4 μM forward primer, 0.4 μM reverse primer (universal *M* primers, Bm-M-1 and Bm-M-1027R [5]; sequences as shown in Table 1) and 0.5 μl of High Fidelity Platinum^®^Taq DNA polymerase (Invitrogen). The PCR amplification cycle was set as 94°C for 2 min for initial denaturation, followed by 35 cycles of 94°C for 30 sec, 55°C for 30 sec, and 68°C for 90 sec, and followed by final extension at 68°C for 10 min. The PCR product of complete *M* segment of 1,056 base pairs in size was gel-purified and cloned into pGEM^® ^T-Easy plasmid (Promega Corporation, Madison, WI). Thereafter, *M* RNA was *in vitro*-transcribed from the recombinant plasmid using Riboprobe^® ^combination system-SP6/T7 (Promega), followed by step of RNase-free DNase (Promega) digestion in order to remove out the recombinant plasmid DNA templates. *M* transcripts obtained were kept at -70°C until assayed.

**Table 1:** Sequences of primers and probes for PCR and real time RT-PCR.

Primer and probe	Sequence (5'>3')	Reference
Bm-M-1	TAT TCG TCT CAG GGA GCA AAA GCA GGT AG	Hoffmann E et al.
Bm-M-1027R	ATA TCG TCT CGT ATT AGT AGA AAC AAG GTA GTT TTT	Hoffmann E et al.
FluA Forward	GAC CRA TCC TGT CAC CTC TGA C	CDC
FluA Reverse	AGG GCA TTY TGG ACA AAK CGT CTA	CDC
FluA Probe^1^	TGC AGT CCT CGC TCA CTG GGC ACG	CDC
RnaseP Forward	AGA TTT GGA CCT GCG AGC G	CDC
RnaseP Reverse	GAG CGG CTG TCT CCA CAA GT	CDC
RnaseP Probe^1^	TTC TGA CCT GAA GGC TCT GCG CG	CDC

^1^TaqMan^® ^probes are labeled at the 5'-end with the reporter molecule 6-carboxyfluorescein (FAM) and with the quencher, Blackhole Quencher 1 (BHQ1) at the 3'-end.

**Figure 1:**
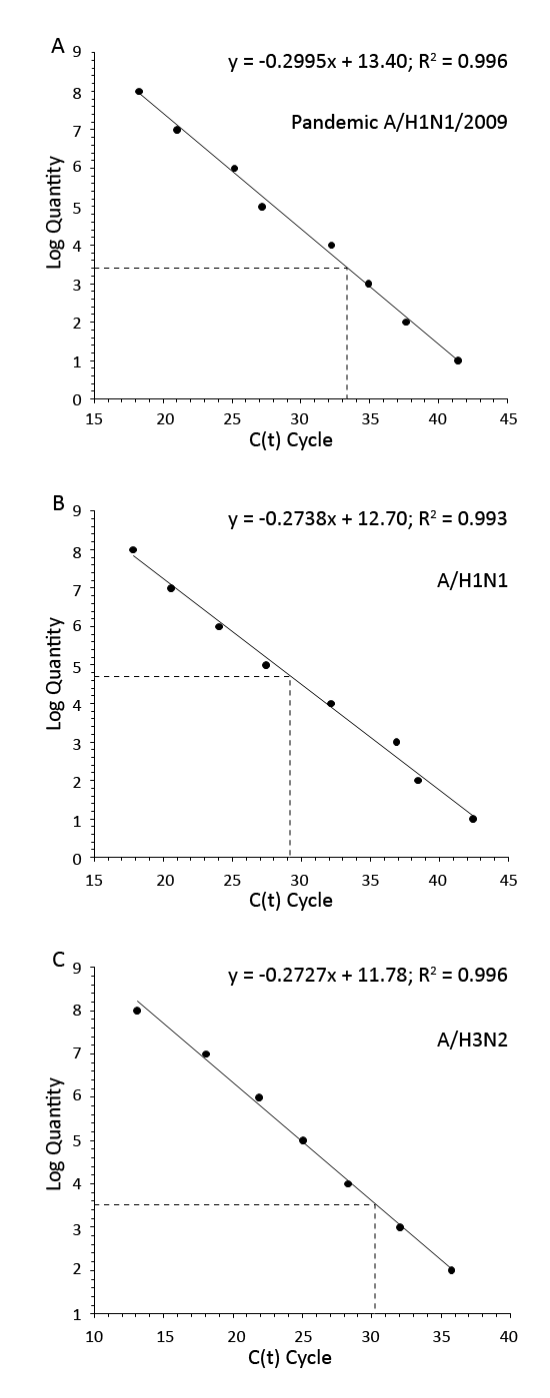
***M *transcript standard curve for quantitative detections of the pandemic A/H1N1 (A), seasonal A/H1N1 (B) and seasonal A/H3N2 viruses (C)**. The standard curve of *M *RNA copy numbers was generated by plotting the Ct value (X-axis) against log_10 _copy numbers of *M *transcripts (Y-axis). The amount of *M *copy number in clinical specimens was obtained by extrapolation of the Ct of the test sample against the standard curve.

To minimize the test variation, standard curves of *M *RNA transcripts were constructed in parallel with the detection of viral *M *RNA in clinical samples in the quantitative real time RT-PCR. The *M *RNA transcripts were measured by Quant-iT™ RNA Assay Kit (Invitrogen) and diluted to various copy numbers in a ten folded serial dilution manner; and each known *M *RNA copy number was assayed by real time RT-PCR according to that described by the 2009 CDC protocol [4]. The sequences of primer and probe sets used in this study are shown in Table 1. A 25 μl reaction mixture of real time RT-PCR comprised 5 μl of total RNA, 12.5 μl of 2× reaction mix, 0.5 μl of SuperScript^TM^ III Platinum^®^Taq Mix (Invitrogen), each 0.8 μM of forward and reverse primers and 0.2 μM of labeled probe, and H_2_O was added to bring up the final volume. The amplification was carried out in DNAEngine^® ^Peltier Thermal Cycler with Chromo4™ Real-Time PCR Detector (Bio-Rad Laboratories, Inc., Hercules, CA) using the amplification cycles of 50°C for 30 min for reverse transcription, 95°C for 2 min for *Taq *polymerase activation, followed by 45 cycles of PCR amplification (95°C for 15 sec and 55°C for 30 sec). Fluorescence signal was obtained at 55°C. The results were analyzed by MJ OpticonMonitor™ Analysis Software version 3.1 (Bio-Rad). A standard curve was constructed by plotting each cycle threshold (Ct) value against the log quantity of standard RNA copy numbers. Total RNA was extracted from the NPA, NS and TS specimens by QIAamp^® ^Viral RNA Mini Kit (QIAGEN Inc., Valencia, CA) following the manufacturer's instruction. Real time RT-PCR for detection of influenza A *M *gene and the *RnaseP *(RNP) house keeping gene, was carried out. To obtain amount of viral load present in each clinical sample, the test Ct value was extrapolated against the standard curve derived from each virus subtype or strain (Fig. 1). The sensitivity of the assay for all 3 subtypes and strain was 100 copies of target *M *RNA/real time RT-PCR reaction when the cut-off for positive result was set at 40 cycles.

### Data Analysis

Statistical analysis was performed with SPSS program. Pair *t*-test was used to compare the mean log_10 _viral loads among different types of specimens collected from the same subjects and at the same time. Student *t*-test was used to analyze the mean log_10 _viral copy numbers in contemporary specimens from patients infected with different virus subtypes and strain.

## Results and Discussion

**Figure 2:**
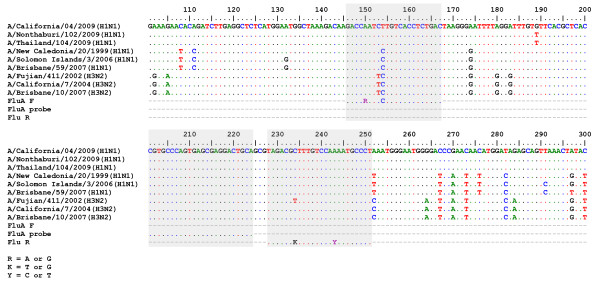
**Alignment of *M *gene fragment from the pandemic A/H1N1, seasonal A/H1N1 and seasonal A/H3N2 viruses against CDC real time RT-PCR primers and probe sequences**. BioEdit Sequence Alignment Editor was used to locate the region of real time RT-PCR primers and probe binding site within *M *gene of various subtypes of influenza A viruses.

Real time RT-PCR protocol was analyzed for its applicability to amplify *M *genes derived from H1N1, H3N2 and the 2009 pandemic viruses by aligning the primers and probe nucleotide sequences against those *M *genes of various influenza subtypes and strains using BioEdit Sequence Alignment Editor (Fig. 2, Table 2). The forward and reverse primers bound to those *M *genes with higher than 90% identity, while the probe bound with 100% identity. This suggested that the CDC primers/probe set can be universally used for detection of *M *segments or viral loads of the novel influenza A/2009 (H1N1), seasonal H1N1 and seasonal H3N2 viruses.

**Table 2:** Percentages of identity of primers and probe with the *M *sequences derived from different virus subtypes and strain.

	% Identity with		
	
Virus	Forward primer	Reverse primer	Probe
Pandemic A/H1N1/2009	95.45	91.67	100
A/H1N1	100	91.67	100
A/H3N2	95.45	91.67-95.83	100

Three standard curves of *M *RNA transcripts were constructed with the R^2 ^of 0.996, 0.993 and 0.996 for pandemic A/2009 (H1N1), seasonal H1N1 and seasonal H3N2, respectively (Fig 1). The *M *copy numbers per ml of VTM from patients infected with pandemic H1N1 or H3N2 viruses were significantly highest in NPA samples (pair *t*-test; *P *≤ 0.05) (Table 3). However, number of patients infected with seasonal H1N1 virus was too small for data analysis. Additionally, viral load levels in patients infected with either subtype or strain was comparable (student *t*-test, *P *> 0.05). *M *RNAs were detected in all NPA and NS, but not in all TS samples collected from patients infected with any one of the virus subtypes/strain. The detection rate was shown in Table 4.

**Table 3:** Influenza viral loads in various types of clinical specimens collected from patients infected with different virus subtypes.

					Log_10 _*M *RNA copy number in		
					
Virus	Number of cases	Statistics	Age	Days after onset	NPA	NS	TS
Pandemic A/H1N1/2009	12	Mean	12	5	7.5^a, b^	6.5^a, c^	4.1^b, c^
		Median	8	5	7.5	6.9	4.9
		Range	3-53	2-10	5.8-8.9	2.7-8.7	Und.-6.9

A/H1N1	5	Mean	5	5	7.8	7.2	7.4
		Median	4	4	8.5	6.4	7.7
		Range	1-12	2-10	4.7-8.9	5.5-9.3	5.8-8.7

A/H3N2	12	Mean	17	5	8.0^a, b^	6.6^a^	5.6^b^
		Median	4	5	8.1	7.2	6.8
		Range	1-69	2-6	5.8-9.2	3.5-8.3	Und.-7.7

The viral loads are reported as log_10 _of *M *segment copy number/1 ml of VTM. Pair *t*-test was used to compare the mean log_10 _viral loads in different types of specimens collected from the same subjects and at the same time.

^a ^indicates a significant difference of the viral loads in NPA and NS, ^b ^in NPA and TS and ^c ^in NS and TS (Pair *t*-test, *P *< 0.05).

Und., below detection limit; NPA, Nasopharyngeal aspirate; NS, Nasal swab; TS, Throat swab.

**Table 4:** Genome detection rate by type of clinical specimens.

	Number of positive cases		
	
Virus	NPA	NS	TS
Pandemic A/H1N/2009	12 (100%)	12 (100%)	9 (75%)
A/H1N1	5 (100%)	5 (100%)	5 (100%)
A/H3N2	12 (100%)	12 (100%)	11 (91.67%)

NPA, Nasopharyngeal aspirate; NS, Nasal swab; TS, Throat swab.

RT-PCR for diagnosis of influenza viruses is generally more sensitive than viral isolation method. The technique detected the viral genome present in dead and alive viruses including excess viral RNA present in the infected cells; however, virus isolation detected only live virus particles. RT-PCR is a high through-put and less time consuming method. In addition, only RT-PCR can differentiate type, subtype and strain of influenza viruses. Sensitivity of RT-PCR to diagnose the disease not only depends on the protocol, but also the type of clinical sample used in the diagnosis. Our study has two advantages that are not commonly conducted in previous reports. Firstly, we had an opportunity to investigate 3 types of clinical specimens collected from the same individuals at the same time, e.g., NPA, NS and TS. Secondly, we had employed full length *M *RNA transcripts derived from A/H1N1, A/H3N2 and the 2009 pandemic viruses to construct 3 standard curves for quantifying viral RNA copy numbers of the contemporary subtype and strain present in the test specimens, with the assumption that the full length *in vitro M *RNA transcripts closely mimics the native structure of the viral *M *genomic segments. Regardless of viral subtypes and strains (H1N1, H3N2 and 2009 pandemic H1N1 virus), we found that all NPA and NS specimens were positive for viral genome detection, while the positive rate was lower in TS specimens.

Previous investigators reported that viral RNA concentration in respiratory samples and long duration of virus shedding were correlated with influenza disease severity [6]. Amount and duration of viral shedding are important in the disease treatment and control of virus spread. Different type of specimens contained different amount of viral RNA concentration; therefore, using different type of clinical specimens may yield different information. In addition, there is no reference method for viral load assay. Peiris et al. [7] reported that viral load in NPA samples of H5N1 patients was lower than those of H3N2 patients. The finding was further extended by Ward et al. [8] that viral load in throat swab samples of H5N1 patients in 1997 and 2004 was 10-fold lower than that observed in H3N2 patients, i.e., 1.5 × 10^6 ^TCID_50_/ml versus 1.6 × 10^5 ^TCID_50_/ml (*t*-test, *P *< 0.05). On the other hand, de Jong et al. [9] found that viral load in TS from H5N1 patients was significantly higher than that from H3/H1 patients; and, additionally, TS contained significantly higher H5N1 viral load than nasal swab samples; meanwhile, viral load in TS and nasal swab samples from H1/H3 patients was not statistically different. The difference in results obtained from different groups of investigators might reflect process of specimen collection and also the different protocols for viral load measurement.

It has been reported that the 2009 pandemic virus preferentially binds sialic acid receptor with α 2, 6 linkage to galactose (SA α 2,6 Gal), the same as human influenza H1N1 and H3N2 viruses [10]. Fatality rate in patients infected with the novel virus is less than 1%, except in that which occurs in patients with underlying conditions, e.g., cardiovascular disease, hypertension, asthma and diabetes, etc. [11,12]. However, the study in a mammalian model demonstrated that the 2009 pandemic H1N1 virus was more pathogenic than the seasonal H1N1 virus [13]. Our study, therefore, explored the viral load in respiratory secretions collected prior to anti-viral treatment, and found that the level of viral RNA in cases infected with the 2009 pandemic H1N1 virus was not statistically different from those infected with seasonal H1N1 and H3N2 viruses. Mean log_10 _copies/ml of viral RNA of 7.5-8.0 in NPA, 6.5-7.2 in NS and 4.1-7.4 in TS samples were found in our study. It is to be kept in mind that all of our patients had severe influenza at time of specimen collection, and most of them were pediatric patients (24 children and 5 adults). Duration of viral shedding of the seasonal influenza as reported by the other groups of investigators was 4-5 days in average [6,14]. A recent report by To et al. [15], showed that the level of the 2009 pandemic viral load of 8 log_10 _copies/ml was found in respiratory specimens collected before oseltamivir treatment; and the viral shedding peaked at the day of onset of symptom with median duration of 4 days [15]. On the other hand, when using plasmid containing amplification target to construct the standard curve together with using pool of throat and nasal swab as the test samples, the other study demonstrated that the H1/H3 viral loads of 5.06 ± 1.85 log_10 _copies/ml were found in patients with major co-morbidities and 3.62 ± 2.13 log_10 _copies/ml in patients without co-morbidities [6].

## Conclusions

Our study suggested that when complete facilities are accessible, such as in clinics and hospitals, NPA will be the best specimen of choice; and in field investigation, NS will be the second choice, followed by TS specimen. Using the appropriate specimen will provide the highest diagnostic rate and the precise strategy for disease treatment and prevention control.

## Competing interests

The authors declare that they have no competing interests.

## Authors' contributions

PP designed the research study; NN, PN, PK and PhP performed research; NN, PN and PK analyzed data; NN and PP wrote the manuscript. KK, TC, CS, CC and JF provided specimens. All authors read and approved the final manuscript.

## References

[B1] Maines TR Szretter KJ Perrone L Belser JA Bright RA Zeng H Tumpey TM Katz JM Pathogenesis of emerging avian influenza viruses in mammals and the host innate immune response Immunol Rev 2008 225 68 84 10.1111/j.1600-065X.2008.00690.x 18837776

[B2] Tognotti E Influenza pandemics: a historical retrospect J Infect Dev Ctries 2009 3 331 334 10.3855/jidc.239 19759501

[B3] WHO information for laboratory diagnosis of pandemic (H1N1) 2009 virus in humans-update http://www.who.int/csr/resources/publications/swineflu/en/

[B4] CDC protocol of realtime RTPCR for influenza A (H1N1) http://www.who.int/csr/resources/publications/swineflu/en/

[B5] Hoffmann E Stech J Guan Y Webster RG Perez DR Universal primer set for the full-length amplification of all influenza A viruses Arch Virol 2001 146 2275 2289 10.1007/s007050170002 11811679

[B6] Lee N Chan PK Hui DS Rainer TH Wong E Choi KW Lui GC Wong BC Wong RY Lam WY Chu IM Lai RW Cockram CS Sung JJ Viral loads and duration of viral shedding in adult patients hospitalized with influenza J Infect Dis 2009 200 492 500 10.1086/600383 19591575 PMC7110250

[B7] Peiris JS Yu WC Leung CW Cheung CY Ng WF Nicholls JM Ng TK Chan KH Lai ST Lim WL Yuen KY Guan Y Re-emergence of fatal human influenza A subtype H5N1 disease Lancet 2004 363 617 619 10.1016/S0140-6736(04)15595-5 14987888 PMC7112424

[B8] Ward CL Dempsey MH Ring CJ Kempson RE Zhang L Gor D Snowden BW Tisdale M Design and performance testing of quantitative real time PCR assays for influenza A and B viral load measurement J Clin Virol 2004 29 179 188 10.1016/S1386-6532(03)00122-7 14962787 PMC7128145

[B9] de Jong MD Simmons CP Thanh TT Hien VM Smith GJ Chau TN Hoang DM Chau NV Khanh TH Dong VC Qui PT Cam BV Ha do Q Guan Y Peiris JS Chinh NT Hien TT Farrar J Fatal outcome of human influenza A (H5N1) is associated with high viral load and hypercytokinemia Nat Med 2006 12 1203 1207 10.1038/nm1477 16964257 PMC4333202

[B10] Neumann G Noda T Kawaoka Y Emergence and pandemic potential of swine-origin H1N1 influenza virus Nature 2009 459 931 939 10.1038/nature08157 19525932 PMC2873852

[B11] WHO, Pandemic (H1N1) 2009 - update 58, Laboratory-confirmed cases of pandemic (H1N1) 2009 as officially reported to WHO by States Parties to the International Health Regulations (2005) http://www.who.int/csr/don/2009_07_06/en/

[B12] WHO, Assessing the severity of an influenza pandemic http://www.who.int/csr/disease/swineflu/assess/disease_swineflu_assess_20090511/19480086

[B13] Itoh Y Shinya K Kiso M Watanabe T Sakoda Y Hatta M Muramoto Y Tamura D Sakai-Tagawa Y Noda T Sakabe S Imai M Hatta Y Watanabe S Li C Yamada S Fujii K Murakami S Imai H Kakugawa S Ito M Takano R Iwatsuki-Horimoto K Shimojima M Horimoto T Goto H Takahashi K Makino A Ishigaki H Nakayama M Okamatsu M Takahashi K Warshauer D Shult PA Saito R Suzuki H Furuta Y Yamashita M Mitamura K Nakano K Nakamura M Brockman-Schneider R Mitamura H Yamazaki M Sugaya N Suresh M Ozawa M Neumann G Gern J Kida H Ogasawara K Kawaoka Y *In vitro *and *in vivo *characterization of new swine-origin H1N1 influenza viruses Nature 2009 460 1021 1025 19672242 10.1038/nature08260 PMC2748827

[B14] Ison MG Influenza in hospitalized adults: gaining insight into a significant problem J Infect Dis 2009 200 485 488 10.1086/600384 19591578

[B15] To KK Chan KH Li IW Tsang TY Tse H Chan JF Hung IF Lai ST Leung CW Kwan YW Lau YL Ng TK Cheng VC Peiris JS Yuen KY Viral load in patients infected with pandemic H1N1 2009 influenza A virus J Med Virol 2010 82 1 7 10.1002/jmv.21664 19950247 PMC7167040

